# Nanoparticulate Drug Delivery Strategies to Address Intestinal Cytochrome P450 CYP3A4 Metabolism towards Personalized Medicine

**DOI:** 10.3390/pharmaceutics13081261

**Published:** 2021-08-16

**Authors:** Rui Xue Zhang, Ken Dong, Zhigao Wang, Ruimin Miao, Weijia Lu, Xiao Yu Wu

**Affiliations:** 1 Institute of Medical Research, Northwestern Polytechnical University, 127 West Youyi Road, Xi’an 710072, China; zhangruixue@nwpu.edu.cn (R.X.Z.); miaomia@mail.nwpu.edu.cn (R.M.); 2595lwj@mail.nwpu.edu.cn (W.L.); 2 Advanced Pharmaceutics & Drug Delivery Laboratory, Leslie Dan Faculty of Pharmacy, University of Toronto, 144 College Street, Toronto, ON M5S 3M2, Canada; ken.dong@alum.utoronto.ca; 3 College of Food Science and Engineering, Nanjing University of Finance and Economics, Nanjing 210003, China; zhigaowang1@gmail.com

**Keywords:** oral drug delivery, drug-drug interaction, bioavailability, CYP3A4, BDDCS, gastrointestinal tract, P-glycoprotein, biological barrier, nutraceutics, lipid-based nanoparticles

## Abstract

Drug dosing in clinical practice, which determines optimal efficacy, toxicity or ineffectiveness, is critical to patients’ outcomes. However, many orally administered therapeutic drugs are susceptible to biotransformation by a group of important oxidative enzymes, known as cytochrome P450s (CYPs). In particular, CYP3A4 is a low specificity isoenzyme of the CYPs family, which contributes to the metabolism of approximately 50% of all marketed drugs. Induction or inhibition of CYP3A4 activity results in the varied oral bioavailability and unwanted drug-drug, drug-food, and drug-herb interactions. This review explores the need for addressing intestinal CYP3A4 metabolism and investigates the opportunities to incorporate lipid-based oral drug delivery to enable precise dosing. A variety of lipid- and lipid-polymer hybrid-nanoparticles are highlighted to improve drug bioavailability. These drug carriers are designed to target different intestinal regions, including (1) local saturation or inhibition of CYP3A4 activity at duodenum and proximal jejunum; (2) CYP3A4 bypass via lymphatic absorption; (3) pH-responsive drug release or vitamin-B_12_ targeted cellular uptake in the distal intestine. Exploitation of lipidic nanosystems not only revives drugs removed from clinical practice due to serious drug-drug interactions, but also provide alternative approaches to reduce pharmacokinetic variability.

## 1. Introduction

Failed drug therapy due to unintended drug-drug interactions occurs widely in clinical medicine and could impact drug efficacy and safety [1,2,3]. According to the World Health Organization, the annual financial cost of medically-related harm globally is approximately $42 billion USD [4]. In order to exert a therapeutic effect, drugs must be absorbed via a certain route of administration, such as oral, intravenous, intramuscular, nasal and subcutaneous, among which oral delivery is the most preferred method due to several well-recognized reasons, including convenience, non-invasiveness, extended drug release, suitability for long-acting medication and a long shelf-life [5,6], but the liver and small intestine constitute the main sites of drug metabolism, leading to pharmacokinetic (PK) variability (i.e., different drug concentrations in the blood or at sites of action) [7,8]. Special populations, such as the elderly, children, women, pregnancy, hospitalized patients and even certain ethnicities, are particularly vulnerable to certain prescribed oral medication(s), because their physiological differences (e.g., metabolism) and/or ongoing life circumstances (e.g., polypharmacy, comorbidities) may result in unpredictable drug-drug interactions [9].

**Figure 1:**
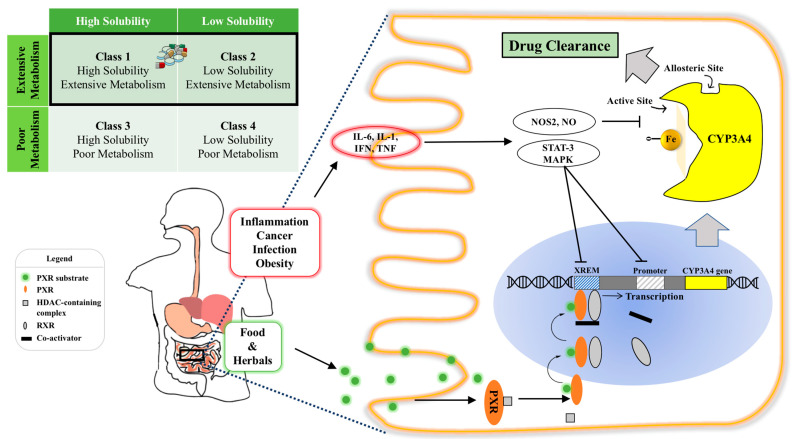
Illustration of intestinal CYP3A4 regulation and the effect of its content and activity variation on orally administered drugs. Top left: CYP3A4 ligands belong to BDDCS Class 1 and Class 2 with varied solubilities and extensive metabolism, highlighted by the black rectangle; Bottom panel: intra-enterocytic CYP3A4 regulation by endogenous factors under disease conditions and xenobiotics from oral intake (e.g., drugs or food constituents). Normally, a PXR ligand enters the enterocyte and binds to PXR intracellularly. This then dimerizes with retinoid X receptor (RXR) and binds to the xenobiotic response enhancer module (XREM) to upregulate the CYP3A4 gene. Systemic inflammatory conditions such as cancer, infection and obesity increase circulating cytokines, such as IL-6, which activate the STAT3-MAPK pathway to downregulate CYP3A4 gene regulation. Intra- and inter-individual CYP3A4 variation can cause varied drug clearance, resulting in undesirable toxicity and ineffective therapy of drugs with a narrow therapeutic index. Abbreviation: RXR, retinoid X receptor; XREM, xenobiotic response enhancer module (XREM); STAT: signal transducer and activator of transcription-3; MAPK: a family of signaling cascade including Jun N-terminal kinase and mitogen-activated protein kinase 1 pathways.

Before reaching the target site of action, orally administered drugs encounter a host of obstacles in the gastrointestinal tract (GIT), such as a substantially changing pH in the stomach, upper and lower intestinal segments, extensive enzymatic degradation (e.g., lipase, trypsin, amylase), varied GI motility (e.g., gastric emptying, peristalsis), complex bacterial diversity and physical barriers of the mucus and mucosal layers [10,11]. Among those complex GIT environmental factors, pre-systemic metabolism of Cytochrome P450 (CYPs) enzymes expressed in the intestine and liver is the main factor that significantly contributes to the variability in drug response [12]. Particularly, out of all the CYPs involved in human drug biotransformation, CYP3A4 is the most important oxidation enzyme by virtue of the fact that at least 50% of marketed drugs metabolized by CYPs are metabolized by CYP3A4 [8,13]. The amount and activity of CYP3A4 is considerably regulated by inflammation, fasting state and a broad spectrum of xenobiotics, including top prescribed pharmaceuticals (e.g., midazolam, felodipine), common foods (e.g., grapefruit juice) and widely used herbal medicines (e.g., St. John wort (SJW)) [14,15,16] (Figure 1). However, drug development selects the most effective and safe dose in the study population (not the individual) without any regard for such drug response variability due to CYP3A4 metabolism, as it is impractical to investigate many different doses in different patients. Yet, from a clinical medicine point of view, “one-dose-fits-all” regimen can be potentially dangerous for patients as huge inter- and intra-individual variability in CYP3A4 may lead to varied systemic drug concentrations, in turn, causing unpredictable therapeutic outcomes and intolerable adverse effects [17].

Despite incongruity between industrial and clinical sectors in how to determine drug dosing, for most drugs, the prediction of their in vivo efficacy and toxicity relies on free (i.e., bioavailable) drug concentration at the site of action [18]. However, before reaching systemic circulation, the passage of orally administered drugs through the GIT could be attenuated by the presence of drug transporters (e.g., P-glycoprotein (P-gp)) and metabolizing enzymes (e.g., CYP3A4) in the small intestine. The additional barrier of first-pass metabolism, as the portal vein flows through the liver, also contributes to drug elimination.

Once systemically available, drugs may partition into other tissues such as the lung and heart, depending on their physiochemical properties such as lipophilicity. In addition, food intake has a strong effect on the extent of oral drug absorption [19,20,21]. Particularly, postprandial state with a high fat meal can have both positive and negative impact on the drug bioavailability by altering the dissolution, the rate and extent of absorption [22]. Ingested dietary lipids (e.g., triglyceride, monoglyceride, fatty acids) are known to increase dissolution and solubilization of lipophilic compounds with log P > 5, modulate GIT transit and inhibit CYPs activity [22,23]. Because of this, the U.S. Food and Drug Administration (FDA) has issued guidance for “Food-Effect Bioavailability and Fed Bioequivalence Studies” to label some medications that are strongly influenced by a meal [24]. All of these factors could confound the predication of a therapeutically relevant dose, and partially attribute to the decline in accumulative clinical success rate (i.e., ~11.6%) [25,26]. As a result, the question of whether adequate drug concentrations at the target have been achieved may underpin variability in drug response.

The influence of those physiological GIT barriers (e.g., CYP3A4, pH) as well as extrinsic regulatory variables (e.g., diet, health conditions) on absorbed drug concentrations can be largely determined by the properties of the oral dosage form, which in turn depends on its design and manufacture [27]. For example, in a randomized crossover study, Mueller et al., compared two marketed cyclosporin formulations, Sandimmune vs. Sandimmune Neoral**^®^**, and found that the long chain triglyceride-based Neoral**^®^** was much less influenced by fat-rich meals, indicating that lipid-based formulations to deliver the drug was advantageous when individualizing a dosage regimen [28,29]. Especially, lipid-based formulations, which account for approximately 2–4% of the pharmaceutical market, represent one of the most popular approaches to improve the solubilization of poorly water-soluble compounds and to overcome the GIT barriers. Pharmaceutical grade lipid excipients in lipid-based formulations are physiological or physiologically related, including fatty acids (e.g., oleic acid, myristic acid), ethyl esters (e.g., ethyl oleate), triglycerides of long- or medium- chain fatty acids (e.g., corn oil, Miglyol**^®^**), and non-digestible mineral oil [30]. The intraluminal process (e.g., bile salts) of ingested lipids from formulations forms solubilised phases to facilitate the drug absorption [30,31]. 

Among diverse lipid-based formulations, lipid-based nanosystems (LNS) have been an ever-growing segment of pharmaceutics that primarily involves FDA’s Biopharmaceutics Classification System (BCS) Class 2 & 4 drugs with low solubility and/or poor permeability [32]. LNS encompasses various subtypes with different nanostructures, including solid lipid nanoparticles (SLN), liposomes, nanostructured lipid carriers (NLC), polymer-lipid hybrid nanoparticles (PLN), and self-nanoemulsifying drug delivery systems (SNEDDS) [33,34,35,36,37]. Because of combined attributes of both lipidic carriers and nanosized particles, LNS can facilitate the delivery of active pharmaceutical ingredients (API) into the blood circulation via either the hepatic portal vein or the GIT lymphatic system [38]. Particularly, for LNS containing triglycerides, cholesterol esters or long chain fatty acids, they are incorporated into the large lipoprotein chylomicrons (75–1200 nm) upon re-esterification and preferentially trafficked via the intestinal lymphatic system, thus effectively evading first-pass metabolism in the liver [39]. In recent years, an increasing body of evidence has shown that certain components (e.g., surfactants, lipids and polymers) used in LNS can reduce intra-enterocyte metabolism [40]. Using CYP3A4 substrate midazolam as the probe, studies of effects of common surfactants, co-solvents and oils (e.g., Tween, PEG, poloxamer, oleic acid) on the drug disposition found 68.2% of tested excipients significantly inhibited CYP3A4 biotransformation activities [41,42]. In addition, with advancing nanotechnology, LNS can be adapted by modulating their lipid composition and decorating them with polymers or ligands to target different regions of the GIT, in order to achieve a controlled, sustained, and targeted drug delivery [43]. 

In this review, we explore the essentiality of overcoming intestinal CYP3A4 as a critical physiological barrier in the GIT. Then, we have selected orally administered drugs using Benet’s Biopharmaceutics Drug Disposition Classification System (BDDCS) and daily dietary constituents or herbal extracts that involve CYP3A4 interactions, respectively. We highlight lipid-based drug delivery strategies that could potentially modulate or bypass intestinal CYP3A4 metabolism. With the aid of computer modeling and advanced micro- and nanotechnologies, LNS with controlled drug release kinetics may potentially tackle in vivo variability of CYP3A4 susceptible drugs with a narrow therapeutic index.

## 2. Intestinal CYP3A4 as a Critical GIT Barrier

### 2.1. Contribution of Gut Wall to Drug Metabolism

CYP3A4 is prominently expressed in both the liver and small intestines, where the total amount of intestinal CYP3A4 content averages about 40% of the liver content [8,44]. As such, it was previously thought that the liver has a greater role in the overall first-pass metabolism of many drugs. However, studies in both humans and animals have suggested the substantial contribution of intestinal CYP3A4 to primary metabolism of drugs and the spatially independent regulation of intestinal CYP3A4 activity from hepatic ones [45,46,47]. In paired human liver and small intestinal specimens, the content of enterocyte CYP3A4 and drug efflux transporter P-gp were three times and seven times, respectively, greater than hepatocytes, and there was a good positive correlation between CYP3A4 intestinal expression and catalytic activity, such as the maximum rate of metabolism (Vmax) of its substrate drug (i.e., verapamil) [47]. 

The selective expression of CYP3A4 in mature enterocytes at the tips of villi mostly in the duodenum and proximal jejunum is evolutionarily strategic, as this is the first-line gating mechanism to prevent absorption of harmful xenobiotics in the body. Yet, the oral bioavailability of several clinically used drugs are limited by intestinal CYP3A4 metabolism, including hydroxylmethylglutaryl coenzyme A (HMG-CoA) reductase inhibitors, calcium channel blockers and benzodiazepines [46,48]. In transgenic knockout CYP3A4 -/- mice in either the intestine or the liver, expression of CYP3A4 in the intestine virtually blocked absorption of orally administered chemotherapeutic drug docetaxel, whereas hepatic expression facilitated systemic drug clearance [49]. 

### 2.2. Drug Metabolism Variation by Enterocytic CYP3A4

In humans, there are huge intra- and inter-individual variations of intestinal CYP3A4 content and activity [50]. Firstly, CYP3A4’s broad specificity is due to its active site ability to simultaneously accommodate molecules of different sizes and kinds within two substrate-binding sub-pockets and one “effector” binding region [51]. CYP3A4 exists in multiple conformations, allowing for high range in activity modulation from effectors (e.g., inhibitors or inducers) in the allosteric sites located close to the active site [51]. For example, one study examined the substrate dependent CYP3A4 inhibition kinetics and found that tested inhibitors exhibited a host of the half maximum inhibitory concentration (IC50) from 0.001 µM to 100 µM depending on the probe substrate used, such as midazolam, testosterone, nifedipine and terfenadine [52]. 

Secondly, although most people follow a trend where CYP3A4 activity decreases along the intestines from the proximal duodenum to the distal ileum, the fold of inter-variability is high. Intestinal CYP3A4 activity from different sections of human donor intestinal tissues using midazolam as a test substrate and its 1’-hydroxy metabolite showed that peak CYP3A4 activity varied as much as 50-fold between each donor [53]. From the analysis of six donor intestines, one showed a near constant CYP3A4 activity, and one showed low proximal activity that spiked in the medial section but reverted back to low distal activity [53]. Because of such CYP3A4 variations at the gut wall, stimuli-responsive (e.g., pH) or sustained dosage forms may exhibit varied pharmacokinetic profiles of systemic drug concentration depending on release location and rate along the GI tract.

Thirdly, CYP3A4’s expression is coordinately regulated by nuclear receptors, particularly pregnane X receptor (PXR) (Figure 1). PXR has the highest mRNA expression in the small intestine and liver, and a broad substrate specificity, including frequently prescribed drugs such as rifampin, phenytoin, phenobarbital, carbamazepine, dexamethasone, paclitaxel, topotecan, and omeprazole [54,55]. Whether or not this broad specificity is the cause of CYP3A4’s own broad specificity remains unclear, but in the same cohort study, CYP3A4 expression was strongly correlated with expression data of PXR [56]. PXR is also downregulated during disease conditions, such as inflammation, cancer, infection and obesity [57]. During inflammation, cytokines [e.g., interleukin-1 (IL-1) or interleukin-6 (IL-6), tumor necrosis factor (TNF)] modulate nuclear receptors, including PXR, in an IL-6 dependent mechanism to downregulate gene expression of CYP3A4 during host defense mechanisms, which leads to potentially increased concentrations of drugs toward toxic levels [14,58].

It is worth noting that genetic factors of CYP3A4 (e.g., nucleotide polymorphisms, mRNA alternative splicing) may not substantially contribute to the pre-metabolism of drug substrate [59]. Unlike other CYPs metabolic enzymes (e.g., CYP2D6) affected by its genetic polymorphisms, large inter-individual variation of CYP3A4 activity exists as a wide Gaussian distribution compared to a classic bimodal distribution of CYP2D6, suggesting that different enzyme isoforms (or variants) of CYP3A4 do not lead to neither poor or extensive metabolizing phenotypes, but rather the variation is most likely due to environmental factors that cause changes in gene expression of CYP3A4 [8].

## 3. Select Compounds Involving CYP3A4 Interaction and Their Classification

The wide substrate variability from the top prescribed pharmaceuticals, herbals and food has made CYP3A4 one of the main causes of metabolic drug-drug interactions in clinical practice (Table 1 and Table 2) [60,61,62,63,64,65,66,67,68,69,70,71,72,73,74,75,76,77,78,79,80,81,82,83,84,85,86,87,88,89,90,91,92]. The domain of the CYP3A4 active site is hydrophobic, thus CYP3A4 prefers large, lipophilic molecules with relatively high log P values greater than 1, indicating these compounds tend to have low aqueous solubility that compromises oral bioavailability [2,93]. According to FDA’s Biopharmaceutics Classification System (BCS), drugs involved in CYP3A4 interaction can fall into any of the categories, for which the majority are in Class 2 with high permeability-low solubility, some in Class 4 with low permeability-low solubility, and a few in Class 1 or 3 with high solubility and varied permeability (Table 1) [60,61,62,63,64,65,66,67,68,69,70,71,72,94]. However, the criteria of BCS (i.e., solubility and permeability) are not sufficiently powered to predict the oral bioavailability of compounds involving CYP3A4 interaction. That means, even for the Class 1 drugs in BCS with well absorbed properties, systemic availability could be low due to the extensive metabolism and clearance by the intestine and liver. 

The systemic bioavailability of drug formulation, termed as F, is defined by the following two equations:

F = (AUC_oral_ × Dose_i.v._)/(AUC_i.v._ × Dose_oral_) (1)

and:

F = f_a_ × (1 − F_TM_) × (1 − E_H_) (2)

where the area under the plasma (blood) curves (AUC) distinguishes between intravenous (i.v.) and oral administration, *f_a_* is the fraction absorbed from the intestinal lumen, F_TM_ is the degree of GI metabolism (or in the lumen), and E_H_ is the hepatic extraction. Benet’s BDDCS takes into account the extent of drug metabolism, and classifies drugs based on the criteria of drug solubility and the intestinal permeability rate: Class 1 high permeability/metabolism and high solubility, Class 2 high permeability/metabolism and low solubility, Class 3 low permeability/metabolism and high solubility and Class 4 low permeability/metabolism and low solubility (Figure 1) [20,95]. Because highly permeable drugs can be reabsorbed from unchanged drug excreting routes and thus can be only cleared by metabolism, it is believed that high intestinal permeability is correlated with extent of drug metabolism [95]. As shown in Table 1, using the BDDCS, most drugs involved in CYP3A4 interactions as substrates, inhibitors and inducers fall into Class 1 and Class 2 with high permeability and metabolism. Especially for Class 2 CYP3A4 substrates in BDDCS with low solubility, these drugs do not achieve adequate concentrations in enterocytes to saturate the enzyme. Increasing the solubility of these substrates by rationally designed dosage forms would overwhelm CYP3A4 so that any pre-systemic metabolism would be negligible (see Section 4 “LNS strategies of overcoming pre-systemic CYP3A4 metabolism”).

CYP3A4 activity is also particularly sensitive to dietary constituents that is related to drug-food and drug-herb interactions, for which several classic reviews have discussed this field of study [96,97]. Table 2 lists common foods and herbals used in the general population that have been shown to inhibit or induce CYP3A4 activity or expression [73,74,75,76,77,78,79,80,81,82,83,84,85,86,87,88,89,90,91,92]. The best-known example is grapefruit-drug interactions that causes marked and variable increases in plasma concentration up to 250% of various orally administered CYP3A4 substrate drugs, including felodipine and nifedipine for treating hypertension, midazolam for anesthesia and cyclosporin for immunosuppression [74,98,99,100,101].

The study of six human subjects with borderline hypertension showed that grapefruit juice, but not orange juice, caused more frequent vasodilatation related adverse effects with felodipine compared to water intake [98]. It was found that the furanocoumarin derivatives from grapefruit juice strongly inhibit the catalytic activity of CYP3A4 in an reversable manner and downregulate intestinal CYP3A4 protein expression by more than 50% [102,103]. Besides traditional foods like fruits, teas and beverages, increasing popularity in complementary alternative medicine (CAM) and concomitant use with medications has resulted in the potential negative clinical consequences of interactions between intestinal CYP3A4 and extracted herbal compounds, such as polyphenols [104]. For example, the widely used herbal St. John’s wort (SJW) for the treatment of depression has been shown to induce the activity of CYP3A4 and P-gp transporter in the intestine [105]. Particularly, SJW extracted hyperforin content of greater than 1 mg daily intake is associated with substantial changes in systemic exposure of drugs, such as digoxin, cyclosporin, and oral contraceptives [106,107]. Therefore, careful consideration must be given to prescribing drugs that involve CYP3A4 metabolism and nutraceutical formulations, where potential dietary isolate constituents affecting pre-systemic metabolism needs to be recognized.

## 4. LNS Strategies of Overcoming Pre-Systemic CYP3A4 Metabolism

**Figure 2:**
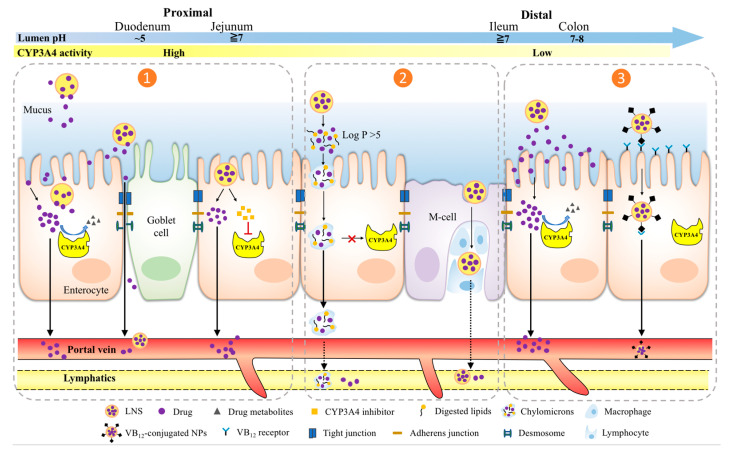
Different absorption strategies of LNS to address drug-response variability by intestinal CYP3A4 metabolism. Left panel (**1**): at the proximal small intestine, intra-enterocytic CYP3A4 activity can be locally saturated or inhibited by LNS delivered API or excipients; Middle panel (**2**): CYP3A4 metabolism is minimized via lymphatic drug transport; Right panel (**3**): Targeting the distal end of the small intestine where CYP3A4 activity is the least. Depending on the approach, various LNS types can be designed, from left to right: Mucoadhesive, CYP3A4 inhibitor-conjugated (combined), Chylomicron-mediated, M-cell mediated, pH-responsive and vitamin B12-mediated.

Many drugs are victims of CYP3A4 interactions. Unfortunately, interactions with CYP3A4 restrict the clinical use for these drugs that ultimately impact patient treatments. If these drugs can be formulated to avoid CYP3A4 binding, then they can be successfully used in therapy. Many lipid-based, polymeric and inorganic drug carriers have been used clinically and experimentally with great success to overcome the limitation of free therapeutics (e.g., solubility) and heterogenous biological barriers (e.g., systemically, microenvironmentally, and cellularly) across patient populations and diseases because of their capability of being engineered in a more personalized manner [108]. Figure 2 illustrates three potential approaches of orally administered LNS to address enterocytic CYP3A4 metabolism and in turn, drug-response variability. 

In Table 3, select LNS examples and their delivered drugs based on BDDCS class are presented to demonstrate their mechanistic strategies of improving oral bioavailability [109,110,111,112,113,114,115,116,117,118,119,120,121,122,123,124,125,126,127,128,129]. By taking advantage of existing physiological characteristics of the small intestines, the following six strategies have the potential to use nanoparticle (NPs) formulations to address intestinal CYP3A4 metabolism and to enhance the absorption of drugs and NPs across enterocytes: (1) incorporating mucoadhesive polymers or lipids that are attracted to the unstirred water layer adjacent to the intestinal epithelia, allowing drugs to be in close proximity which increases the flux into epithelial cells to overwhelm CYP3A4 metabolism; (2) CYP3A4 inhibitor-containing LNS locally inhibits intestinal CYP3A4 during transcytosis; (3) using highly lipophilic lipid NPs to traverse enterocytes straight into lymphatic vessels; (4) formulating LNS targeting M cell integrins for endocytosis that carries the drug to lymphatic vessels; (5) pH-sensitive formulation that selectively releases drug in the ileum where there is a lower expression of intestinal CYP3A4; (6) vitamin B12 targeting cubilin in the terminal ileum for absorption via receptor mediated endocytosis. Those strategies are GIT region dependent, so we discuss them separately below. 

### 4.1. Local Saturation or Inhibition of Enterocytic CYP3A4 Activity at Proximal GIT

The duodenum and proximal jejunum are the main absorptive sites of designed lipophilic drug formulations due to the different morphology and mucosal cell differentiation [130]. First, the thickness of adherent mucus layers of proximal GIT is the thinnest among other regions (e.g., stomach, ileum, colon); in humans, the small intestine mucus layer has a uniform thickness of 15.5 µm compared to 135 µm in the colon [131,132]. Also, pH is substantially increased from the extremely acidic stomach (~0.8–5) to less acidic duodenum (~7) [132]. As a result, API from pH-responsive formulations are often locally released at high luminal concentrations. This increases the concentration at the cell surface and could potentially increase the transport gradient into enterocytes according to Fick’s first law of diffusion, which states that diffusive flux into a compartment is proportional to the concentration gradient in a linear dimension. Mathematically, this can be expressed as:

J ∝ −∂C/∂x(3)

where J is the diffusion influx, C is the concentration of substance, and x is the thickness or length. Since CYP3A4 activity is the highest in the duodenum, increasing the intracellular concentration of drug substrate would saturate and overwhelm drug metabolizing enzyme CYP3A4 to improve absorption (Figure 2). Yet, because of rapid proximal GIT peristalsis from oral to anal side, for drugs to be locally absorbed, incorporation of mucoadhesive polymers is sometimes required to prolong residence time of LNS at this region [133,134]. Once adhered to the mucin or epithelial surface, the rate of drug release in vivo depends on the chemical composition of LNS (Table 3). In a study of an oral formulation of SLN encapsulating cyclosporin A, the mixture of the stabilizer, such as lecithin or sodium cholate, of the SLN resulted in fast degradation and drug release, while using poloxamer caused steric hinderance towards epithelial adsorption that resulted in slower degradation and drug release [109,135,136]. It was expected that an intermediate release profile can be achieved by mixing the proportion of lecithin and poloxamer in the stabilizing layer of SLN. In addition, not only used as mucoadhesive and carrier components, some polymers and fatty acids (e.g., different chain length) also serve as permeability enhancers to influence regulation of intestinal tight junctions. As a result, both released and encapsulated drugs are absorbed via paracellular route with transiently opened tight junctions, which in turn avoids intra-enterocyte CYP3A4 metabolism [137,138]. For example, enhancing NPs adsorption at the mucus layer, surface coating of liposomes with the cationic polysaccharide chitosan can electrostatically interact with negatively charged mucin secreted from small intestines [121]. When chitosan was thiolated (known as thiomers), the thiol group can reversibly open gap junctions, as shown using the thiomer matrix system [139]. 

The use of small molecule enzyme inhibitors, co-administered with CYP3A4 susceptible drugs, remains problematic as these agents have a toxic potential caused by the inhibition of physiological enzyme activities [137]. So far, the study of LNS delivery with respect to CYP3A4 inhibition is limited but applying polymeric nanoformulations to resolve the toxicity issue of enzyme inhibition is promising [140,141]. For example, encapsulation of a CYP3A4 inhibitor (i.e., ritonavir) in solid drug nanoformulations can lower the cytotoxicity of inhibitors and also enhance CYP3A4 inhibition and permeability across intestinal Caco-2 cells [141]. To allow transcellular delivery of susceptible drugs by LNS into the systemic circulation without being subjected to CYP3A4 metabolism, the more practical strategy is to select pharmaceutical excipients known for causing induction or inhibition of CYP3A4 (Figure 2 left panel), although their extent is not as significant as known enzyme inhibitors. Readers could refer to the recent review for effects of pharmaceutical excipients on drug metabolism [40]. The most potent inhibitors of CYP3A4 are surfactants and the least effective ones are organic solvents. Tompkins et al., has shown that polysorbate-80 (PS-80), which is a surfactant, inhibited CYP3A4 activity by 70% [142]. The authors believe that its mechanism of CYP3A4 inhibition is probably through extracellular membrane signaling since PS-80 is unlikely to cross the cell membrane due to its high molecular weight and polarity. This type of interaction can be used in future studies as a milder form of bypassing CYP3A4 enzymes without using highly potent inhibitors such as ritonavir, to avoid complicating drug-drug interactions. Drug-excipient interactions also have the added benefit of the excipients being pharmacologically inert, so patients would not experience unnecessary side-effects. This method can be combined with mucoadhesive systems as described above to further improve bioavailability.

### 4.2. Minimizing CYP3A4 Drug Metabolism via Intestinal Lymphatic Drug Transport

Highly lipophilic LNS, particularly containing triglycerides, are degraded through lipolysis in the intestinal lumen and further absorbed by enterocytes for re-assembly into chylomicron intracellularly. Because of the leaky nature of the lymphatic capillaries, drug-incorporating chylomicrons are secreted into the mesenteric lymph and preferentially shunted into the lymphatic vessel instead of the portal vein (Figure 2 middle panel). The major benefit of such delivery approach for the absorption of drugs is avoiding hepatic first-pass metabolism and targeting lymphatic regions of intestine. In order to use this approach for enhanced drug absorption, drugs must have a large log P value (preferably log P > 5) (e.g., efavirenz and atorvastatin in Table 3), have a lipid solubility >50 mg/g, and must be encapsulated or co-ingested with lipids [143]. Ingested lipids from high-fat meals or formulations stimulate the packaging of the drug into chylomicrons for lymphatic transport. Furthermore, a higher intracellular lipid load in enterocytes may form larger lipid droplets that reduces access for the lipid-dissolved drug into metabolizing enzymes (e.g., CYP3A4) [143]. Drugs of interest can be modified by attaching fatty acids to mimic the lipid absorptive process. Alternatively, lipophilic side groups or ester linkages can increase the lipophilicity of the drug to stimulate lymphatic absorption [144]. Furthermore, there have been some studies that use unsaturated fatty acid formulations to enhance absorption, and it is believed that the unsaturated fatty acids compartmentalize in and disrupt the phospholipid bilayer’s interior by interacting with the polar head of phospholipids [145,146,147]. The lipid NPs protect CYP3A4 susceptible drugs from pre-systemic metabolism without releasing them during transcytosis (Table 3). It is worth noting that blocking chylomicron formation by cycloheximide in vivo noticeably reduced AUC of plasma drug concentration, but the lipid NP can transverse enterocytes into the portal vein to compensate the lymphatic blockage [119].

Adapted lipid NPs with surface modification of polymers, such as PLN, can enter intestinal lymphatics via microfold (M) cells. There are regions of gut-associated lymphoid tissue in the intestines called Peyer’s patches, and these are localized mainly in the ileum where protection from bacteria in the large intestines is needed. M cells located in the Peyer’s patches are specialized cells that non-specifically endocytose luminal fluid for the presence of antigens [148]. Because these cells have a high propensity for endocytosis, this could be a method to transport protein-based macromolecules (e.g., antigen, insulin, erythropoietin, growth hormone and clotting factors) that are degraded by proteases in the GI lumen or are too large to diffuse through the intestinal membrane [149,150]. Because the M cells can transport antigens into the immune-inductive environment of the Peyer’s patches (Figure 2 middle panel), oral immunization using NPs entrapped with the whole virus has been developed, such as an oral vaccine for pertussis used to prevent whooping cough [150,151]. However, exploitation of the potential of M cell uptake and transport by LNS can be extended to include any drugs that have extensive pre-systemic and first-pass metabolism. Because of the large proportion of drugs that are metabolized by CYP3A4, any mechanism that avoids this enzyme could be beneficial to avoid drug interactions and other adverse drug reactions.

Strategies to target M cell delivery have been designed by mimicking the entry of pathogens (e.g., *Yersinia, Salmonella*, and *Shigella*) into these cells, or targeting specific receptors (e.g., integrins) on the apical surface of M cells for internalization. Polyethylene glycol (PEG) is a useful stabilizer for NPs because it prevents interactions with macrophages and prolongs the residence time of drug in the body. This can be used with covalently attached RGD (arginine-glycine-aspartate) derivatives to target M cell integrins for internalization [152]. Alternatively, studies on NPs conjugating with *Salmonella* extract or *Yersinia* adhesin (i.e., invasin) have shown increased intestinal absorption into M cells [153,154]. One of the drawbacks for this strategy of drug absorption is that M cells only comprise of 1% of the total intestinal surface, making the therapeutic exploitation of this route probably unrealistic [151,152]. Multifunctional LNS with uptake across absorptive enterocytes and paracellular junction is likely to be needed to complement the systemic drug delivery. For example, a PLN surface modified with positively charged hydroxypropyl trimethylammonium chloride chitosan is designed to take multiple pathways for delivery of docetaxel, a BDDCS Class 2 drug with extensive metabolism (Table 3) [123]. In both an in vitro follicle-associated epithelium (FAE) model and in vivo examination of Peyer’s patches, PLN had high accumulation, suggesting extensive phagocytosis of M cells in addition to tight junction opening and enterocyte endocytosis.

### 4.3. Targeting Distal GIT to Exploit the Least CYP3A4 Activity

The distal jejunum and ileum represent another spatiotemporal target for LNS in the GIT to improve oral drug bioavailability. Physiologically, the CYP3A4 activity in this region is not as high as the proximal segments, indicating lower drug metabolism, and a distinct pH range exists: in typical healthy individuals, the pH of the stomach is 2, the pH of the duodenum ranges from 2.4 in the proximal duodenum to 6.8 in the distal duodenum, the pH of the jejunum ranges between 6 and 7, and the pH of the ileum is approximately 8. These characteristics can be exploited during drug delivery if a drug formulation releases its drug at a specific pH. Most CYP3A4 substrates and modulators are lipophilic (Table 1 and Table 2); as a result, these compounds are relatively able to easily traverse the apical membrane into enterocytes, but their low solubility prevents them from achieving a high concentration to overwhelm the enterocyte’s CYP3A4 enzymes. By formulating a pH-sensitive LNS, which typically comprises of a lipidic core for loading drugs and a pH-responsive polymeric shell structure to release the drug specifically in the distal small intestine, a higher concentration can be achieved in the lumen. With the high influx of drug molecules into the enterocyte, the resultant high intracellular concentration of drug would supersaturate and overwhelm intestinal CYP3A4 enzymes causing a greater amount to bypass metabolism (Figure 2 right panel). 

Enteric coating polymers, such as polymethacrylates (Eudragit*^®^*), cellulose esters, and polyvinyl derivatives, have been widely used in the design of pH-sensitive oral dosage forms and delivery systems for drug protection and controlled release [155,156,157]. For example, Eudragit L100-55 is pH-sensitive methacrylic copolymer that has been successfully used in oral nanoformulations to increase the bioavailability of insulin and an experimental HIV protease inhibitor drug [158,159]. The achieved relative bioavailability of 87.4% for the HIV protease inhibitor drug can be explained by an increase in surface area, more rapid dissolution and a greater dispersion in the NP matrix [159]. To leverage the delivery efficiency for drugs with low water solubility and high first-pass metabolism, various monolithic and multiparticulate systems are designed by combining the use of enteric coating and other formulation methods (Table 3) [126]. In the study of oral delivery of BDDCS Class 1 drug irinotecan to the colon tumor, the multiparticulate system was designed to incorporate folic acid grafted SLN into microbeads of alginates coated by a pH-responsive enteric polymer (i.e., Eudragit 5100). The colon tumor accumulation and systemic concentration of SLN were markedly higher in microbeads coupled than non-coupled ones [126].

The distal ileum is characterized with unique receptors (e.g., vitamin B_12_ (VB_12_) receptor) and transporters (e.g., apical sodium-dependent bile acid transporter (ASBT) for bile acid transport), which can be exploited by LNS to avoid extensive CYP3A4 metabolism in the duodenum and jejunum. Most large orally ingested molecules are absorbed in the intestines through specific influx transporters, such as peptide transporter 1 (PEPT1), organic anion transporting polypeptides (OATPs) and monocarboxylate transporters (MCT1) [160]. These molecules may be metabolized in the enterocytes and continue along into the portal vein where they may be subjected to further metabolism in the liver. In two studies of delivering the BDDCS Class 2 drug docetaxel for cancer treatment, targeting highly expressed MCT1 or ASBT using acetic acid or glycocholic acid conjugated chitosan modified liposomes significantly enhanced docetaxel bioavailability compared to non-targeted lipid NPs (Table 3) [127,128]. Alternatively, in the human body, the intricate absorption mechanism of VB_12_ can be exploited to enhance drug absorption. VB_12_ is a fairly large molecule and when ingested, it becomes attached to a gastrically secreted intrinsic factor where it is recognized by cubilin, a VB*_12_* transporter in the terminal ileum to be absorbed by receptor mediated endocytosis [161]. Since most tumor cells have an over-expression of VB_12_ receptors, some drug molecules or polymers are chemically linked to VB_12_, and it has been suggested that conjugation at the 5*′*-hydroxyl moiety of the ribofuranoside of VB_12_ has the least effect on intrinsic factor binding [162]. Although the capacity for humans to absorb VB_12_ is limited to only 1–2 µg per dose [163], increasing the frequency of administration or formulating a sustained-release delivery system can increase absorption since cubilin recycles every 30 min [164]. In addition, a mucus penetrating LNS may be needed to facilitate contacting enterocytes, as the mucus layer in the distal region of intestine is much thicker (9-fold) compared to proximal small intestines. For example, encapsulation of poorly water-soluble curcumin, a CYP3A4 inhibitor, into VB_12_ targeted PLN improved its systemic concentration via multiple cells pathways at the ileum section (Table 3) [129]. 

## 5. Translating LNS for Personalized Medicine 

In precision medicine-relevant applications, engineering intelligent NPs allow for tailored drug therapies to overcome the heterogeneity across patient populations and diseases [108]. Computer modeling has been utilized to determine similarities in substrates, inducers, and inhibitors of CYP3A4 as well as other drug metabolizing enzymes and drug transporters. For example, it is hypothesized that one type of drug efflux transporter, P-gp, may act synergistically with CYP3A4 due to overlapping drug specificity, contributing to low bioavailability of drugs [47]. Computer modeling can aid medicinal chemists and pharmaceutical industries to design drugs that avoid binding to arrays of enzymes and transporters, in turn to minimize drug metabolism and drug interactions. Moreover, oral forms of medications containing “inactive” ingredients are found to be associated with adverse reactions in patients, and the complexity of the formulation landscape requires in-depth understanding of biological interactions [165]. Some commonly used excipients (e.g., lipids, surfactants, polymers) are found to interfere with intestinal metabolism or efflux mechanisms, such as Cremophor RH40 inhibiting both CYP3A4 and P-gp, and Tween-20 and Pluronic P85 increasing the transporter activity of breast cancer resistance protein [166,167]. Using molecular dynamics stimulation, El-Sayed et al., found that the inhibition mechanism of CYP3A4 by NPs is due to blocking the exit channel for substrate products (i.e., testosterone and its metabolite 6β-hydroxy testosterone), and the surface modification of NPs with PEG attenuated the inhibitory capability of NPs [168]. 

Achieving controlled release at targeted GIT regions without drug precipitation is critical for oral solid dosage forms and delivery systems. For LNS, chylomicron or micelles containing drugs may not be formed, as released poorly soluble drugs have a high propensity to precipitate in the gut lumen upon dissolution. Rational combination of LNS with the pharmaceutically prepared controlled release amorphous solid dispersion systems (CRASD) may provide consistent and predictable drug absorption. For example, celecoxib is non-steroidal anti-inflammatory drug taken orally for pain relief, and it belongs to BDDCS Class 2 with low solubility and extensive metabolism. Different CRASD of celecoxib were formulated using the excipients polyvinylpyrrolidone (PVP) and polyvinyl acetate (PVAc) as controlled release agents. Incorporation of PVAc in two commercial forms, Kollidon*^®^* SR powder and Kollicoat*^®^* SR30D aqueous dispersion, showed distinct drug release. Matrix formed granules had rapid drug release but greatest improvement of celecoxib solubility, while membrane-coated beads demonstrated a sustained release thereby greatly alleviating the possibility of precipitation during dissolution [157]. Even for high solubility drugs without concern of precipitation, the capacity of fine-tuning the drug release is desired to protect the drugs from being metabolized by CYP3A4 in enterocytes. Yet, most lipid NPs exhibit the initial burst of drug release and the released drug amount depends on the formulation matrix. Combining the pH-responsive polymers into LNS may alleviate the issue of unwanted drug release. For example, the release of diltiazem, a BDDCS Class 1 drug, from ethylcellulose-coated dosage forms can be fine-tuned at both pH 6.8 (intestine) and pH 1.2 (stomach) by incorporating and adjusting the levels of pH-responsive polymer-based poly(methacrylic acid)-polysorbate 80-grafted-starch terpolymer nanoparticles (TPNs) (i.e., 5%, 10%, 15% TPN) [156]. In addition, food-based polymers have been applied in oral drug delivery due to their GIT friendly property, large production and facile chemical modification. For example, purified rapeseed protein isolate can be biochemically modified in a controllable manner to be amphiphilic and pH-resistant, which can be combined with LNS to deliver various drugs with different physiochemical properties [169,170,171]. These results implicate that combing LNS with proper oral solid dosage forms may potentially resolve the drug release issue encountered by LNS, paving the way for precise delivery of drugs with extensive metabolism. 

Of particular interest is the recent oral delivery of antiviral drugs such as remdesivir or protease inhibitors for treating coronaviruses (COVID-19) or HIV, and nutraceuticals, such as phytochemicals(e.g., ellagic acid, curcumins) [172]. With mild to moderate daily doses, those compounds can substantially interact with CYP3A4, causing severe adverse effects [61,173]. Therefore, LNS can be combined with micro- and nano- device platforms, such as micro-patches or needles and multi-compartment capsules, to deliver these drugs to intestines [132], which in turn may accelerate the clinical translation of LNS to benefit across different patient populations.

## 6. Conclusions

In summary, there are many drug-drug interactions that occur due to the CYPs system. This usually should not pose a major issue because adverse drug reactions usually occur when drug concentrations are very high, very potent inhibitors are used, or when drugs are suicide substrates. However, drug-drug interactions are still occurring, especially in today’s society where people are engaging in polypharmacy. By designing orally administered LNS to address intestinal CYP3A4 metabolism, these strategies have the potential to improve bioavailability and reduce drug-drug interactions for susceptible CYP3A4 drug candidates. Pharmaceutical knowledge in the field of drug metabolism is important to reduce toxicity, reduce drug-drug interactions, and reduce the occurrence of beneficial drugs being removed from the market.

## Figures and Tables

**Table 1:** Clinically used oral drug formulations involving CYP3A4 interaction and their pharmaceutical classification.

Interaction with CYP3A4 ^a^	Drug Examples	Drug Class	Indications	Formulations ^b^	BDDCS Class ^c^	BCS Class ^d^	Log P ^e^
Substrates	Alprazolam	Benzodiazepine	Anxiety disorders and panic disorder	Tablet, concentrated liquid	Class 1	Class 2	2.12
	Atorvastatin	HMG-CoA reductase inhibitor	Hypercholesterolemia	Tablet	Class 2	Class 2	6.36
	Carbamazepine	Anticonvulsant	Seizures, bipolar disorder, trigeminal neuralgia, diabetic neuropathy	Tablet, capsule, suspension	Class 2	Class 2	2.45
	Cyclosporine	Immuno-suppressant	Prevention organ rejection severe psoriasis, severe rheumatoid arthritis	Capsule, solution	Class 2	Class 2	1.4
	Dexamethasone	Steroid derivative	Different inflammatory conditions: allergic disorders, psoriasis, rheumatoid arthritis, ulcerative colitis	Tablet, solution	Class 1	Class 1/Class 3	1.83
	Ethinyl estradiol	Hormone derivative	Contraceptive, menopausal symptoms	Tablet	Class 1	Class 1	3.67
	Felodipine	Calcium channel blocker	Hypertension	Tablet	Class 2	Class 2	3.86
	Indinavir	HIV protease inhibitor	Cocktails for HIV infections	Capsule	Class 2	Class 4	3.49
	Itraconazole	Azole antifungal	Infections caused by fungus, including the lungs, mouth or throat, fingernails.	Capsule, tablet, solution	Class 2	Class 2	5.66
	Lovastatin	HMG-CoA reductase inhibitor	Hypercholesterolemia	Tablet	Class 2	Class 2	4.26
	Ritonavir	HIV protease inhibitor	Cocktails for HIV infections	Capsule, tablet, solution	Class 2	Class 4	6.27
	Saquinavir	HIV protease inhibitor	Cocktails for HIV infections	Capsule, tablet	Class 2	Class 4	4.7
	Sildenafil	cGMP Phosphodiesterase-5 inhibitor	Erectile dysfunction, pulmonary arterial hypertension	Tablet, capsule, suspension	Class 1	Class 2	2.75
	Simvastatin	HMG-CoA reductase inhibitor	Hypercholesterolemia	Tablet, suspension	Class 2	Class 2	4.68
	Tadalafil	cGMP Phosphodiesterase-5 inhibitor	Erectile dysfunction, enlarged prostate	Tablet	Class 2	Class 2	1.42
	Triazolam	Benzodiazepine	Insomnia	Tablet	Class 1	n/a	2.42
	Vardenafil	cGMP Phosphodiesterase-5 inhibitor	Erectile dysfunction, pulmonary arterial hypertension	Tablet	Class 1	Class 2	2.79
	Verapamil	Calcium channel blocker	Hypertension, angina, cardiac arrhythmias	Tablet	Class 1	Class 1	3.79 at pH 9.0; 2.15 at pH 7.0
Inhibitors	Clarithromycin	Macrolide antibiotic	Bacterial infections: stomach ulcers caused by *Helicobacter pylori*	Tablet, suspension	Class 3	Class 2	3.16
	Erythromycin	Macrolide antibiotic	Different types of bacterial infections	Capsule, tablet, liquid	Class 3	Class 3	3.06
	Fluconazole	Azole antifungal	Fungus infections, cryptococcal meningitis	Powder, tablet	Class 3	Class 1	0.5
	Ketoconazole	Azole antifungal	Fungus infections	Tablet	Class 2	Class 2	4.34
	Midazolam	Benzodiazepine	Anesthesia, anxiety, panic disorder, seizures	Syrup	Class 1	Class 1	4.33
	Nicardipine	Calcium channel blocker	Hypertension, angina, cardiac arrhythmias	Capsule	Class 1	Class 2	3.82
	Nifedipine	Calcium channel blocker	Hypertension, angina, cardiac arrhythmias	Tablet	Class 2	Class 2	2.20
	Tacrolimus	Immuno-suppressant	Prevention of graft rejection following solid organ or bone marrow transplantation	Capsule, granule, tablet	Class 2	Class 2	3.03
Inducers	Phenobarbital ^f^	Anticonvulsant	Seizures, sedation, insomnia	Elixir, tablet	Class 1	Class 1	1.47
	Phenytoin	Anticonvulsant	Seizures, arrhythmia	Capsule, tablet, suspension	Class 2	Class 2	2.47

^a^ Effect of each drug on CYP3A4 is obtained from Philip et al., 2015 [60]; ^b^ the indication and formulation of each drug is found in MedlinePlus (https://medlineplus.gov/) and Drugs.com (https://www.drugs.com/); ^c^ BDDCS class for each drug is obtained from Benet et al., 2011 [61]; ^d^ BCS class for each drug is found from the following references [62,63,64,65,66,67,68,69,70,71]; ^e^ the Log P value of each drug is obtained from PubChem (https://pubchem.ncbi.nlm.nih.gov/), and the detailed references are listed in the Supplementary Materials; ^f^ Interactions between Phenobarbital and CYP3A4 was obtained from FDA [72].

**Table 2:** Widely consumed dietary and herbal products that contain CYP3A4 modulating compounds.

Effect on CYP3A4 Activity	Diet & Herbal	Main Use	Modulating Constituent ^a^	References
Inhibition	Grapefruit	Fruit, juice	Bergaptol, flavonoids (naringin, naringenin, kaempferol and quercetin), furanocoumarins (bergamottin and DHB), paradisin C	[73,74]
Inhibition	Seville orange	Fruit, juice, marmalade	DHB, bergamottin,	[75,76]
Inhibition	Red wine	drinks	Polyphenolic (trans-resveratrol), red wine solids (flavonoids and other polyphenols), gallic acid	[77,78]
Inhibition	Garlic	Food, flavoring agent, supplement	Flavonoids (tangeretin, nobiletin, rutin, quercetin), garlic sulfur containing compounds (DADS, DAS, AMS)	[79,80]
Inhibition	Cranberry	Fruit, juice, supplement	Triterpenes (maslinic acid, corosolic acid, and ursolic acid), anthocyanidins, anthocyanins	[81,82]
Inhibition/Induction	St. John’s wort	Herbal/dietary supplement	Hyperforin, hypericin, quercitrin	[83,84]
Inhibition/Induction	Ginkgo biloba	Alternative medicine	Bilobalide, ginkgolide A	[85,86]
Inhibition	Goldenseal	Botanical supplement	Individual isoquinoline alkaloids (berberine, hydrastine)	[87,88]
Inhibition	Green tea	Drinks, dietary supplement	Green tea catechins, EC, EGC, ECG, EGCG	[89,90]
Induction	Ergot alkaloids	Medicine	Ergotamine	[91,92]

^a^ abbreviation: DHB:6′-7′- dihydroxybergamottin; DADS: garlic’s diallyl disulfide; DAS: diallyl sulfide; AMS: allyl methyl sulfide; EC: (-)-epicatechin; EGC: (-)-epigallocatechin; ECG: (-)-epicatechin-3-*O*-gallate; EGCG: epigallocatechin-3-gallate.

**Table 3:** Examples of orally administered LNS formulations for improving drug bioavailability.

LNS ^a^	Delivery Mechanism	Nanoformulations ^b^	Drug Payload	BDDCS Class ^c^	Study Models	Main Effects ^b^	Reference
**Lipid NPs**	Mucoadhesive	SLN	Cyclosporin A	Class 2	Young pig	- low variation in drug bioavailability	[109]
	Mucoadhesive	VP16-NLC	Etoposide	Class 3	Rat intestinal membrane, Healthy rat	- ↑ intestine permeability - ↑ bioavailability by 1.8-fold compared to VP16 suspension	[110]
	Clathrin-mediated endocytosis	DRD-SLN	Dronedarone hydrochloride	Class 2	Healthy rat	- ↑ bioavailability by 2.68-fold compared to DRD suspension - possible transport via lymphatic absorption	[111]
	Lymphatic transport via chylomicrons	EFV-SLN	Efavirenz	Class 2	Chylomicron blocking rat model, Mesenteric lymph duct cannulated rat model	- ↑ drug amount in the liver - ↑ accumulation in the spleen	[112]
	Lymphatic transport via chylomicrons	AT-NLC	Atorvastatin	Class 2	Both High-fat diet treated and health rats	- ↑ bioavailability by 3.6- and 2.1-fold compared to AT suspension and Lipitor^®^ - improved efficacy of AT by reducing serum levels of TC, TG and LDL	[113]
	Portal vein and lymphatic pathway transport	Darunavir-SLN	Darunavir	Class 2	Everted rat intestine, Chylomicron blocking rat model, Healthy rats	- ↑ bioavailability by 2-fold compared to marketed Darunavir tablet - uptake by enterocyte via endocytosis	[114]
	Portal vein and lymphatic pathway transport	AM-SLNs	Asenapine maleate	Class 1	Caco-2 monolayer, Chylomicron blocking rat model	- ↑ bioavailability by 50.19-fold compared to AM dispersion	[115]
	Lymphatic absorption	CLA-SLN	Clarithromycin	Class 3	Healthy rat	- ↑ bioavailability by 5-fold compared to CLA suspension	[116]
	Lymphatic absorption	GEN-loaded SLN	Genistein	CYP3A4 inhibitor	In vitro characterization of chylomicrons Caco-2 cells, Ex vivo porcine duodenum	- 2-fold increase in uptake of intestinal mucosa and enterocyte	[117]
	Lymphatic transport via chylomicrons	micelles	5-demethylnobiletin	N/A	Caco-2 monolayer	- ↑ enterocytic metabolism of 5DN - fatty acid types affected differently on intestinal uptake of the drug and chylomicron formation	[118]
	Portal vein and lymphatic pathway transport	CCN	Candesartan cilexetil	Class 4	Caco-2 monolayer, in situ single-pass intestine perfusion, ligated intestinal loop model, Healthy rats	- internalized into enterocytes by clathrin-mediated endocytosis - ↑ permeability in the duodenum, jejunum and ileum - ↑ plasma AUC by 10-fold than free CC suspension	[119]
**PLN**	Mucus penetration	pSLN	Doxorubicin	Class 1	Caco-2/HT29 co-culture, Everted rat intestine, Intestine loops model, Healthy rat	- ↑ mucus penetration - ↑ bioavailability by 1.99-fold compared to non-PEGylated SLN	[120]
	Mucoadhesive	Chitosan coated liposome	Alendronate	Class 3	Caco-2 monolayer, Healthy rat	- strong adsorption with mucins - ↑ bioavailability by 2.6-fold compared to alendronate solution	[121]
	Enterocyte adhesive via WGA-lectin binding	LPSN	Paclitaxel	Class 2	A549 cells, Healthy rats	- ↑ retention time in blood compared to free PTX - ↑ plasma AUC, peak concentration, *t*_1/2_ compared to free PTX	[122]
	M-cell phagocytosis, TJ opening, and caveola-mediated endocytosis	HACC-DTX-SLN	Docetaxel	Class 2	Caco-2 monolayer, FAE monolayer, Healthy rat	- high Peyer’s patch accumulation - Reversable regulation of tight junction	[123]
	Lymphatic uptake	NCC-SLN	Curcumin	CYP3A4 inhibitor	Healthy rat	- ↑ plasma AUC by 9.5-fold compared to curcumin solution - 6.3-fold higher accumulation in lymph nodes than curcumin solution	[124]
	pH responsive drug release (i.e., pH 1.2 and pH 7.4)	EuC-NLS	Alendronate sodium	Class 3	Healthy rabbit	- ↑ bioavailability by 12-fold than ALS tablets without enteric coating polymer	[125]
	pH-responsive drug release, (i.e., pH>7.0)	IRSLNF3	Irinotecan hydrochloride trihydrate	Class 1	Healthy mice, HT-29 bearing mice	- ↑ 1.62-fold in plasma AUC compared to NPs without coating pH sensitive microbeads - ↑ inhibition of tumor growth	[126]
	Targeting MCT1 transport	DTX-ACSL-Lip	Docetaxel	Class 2	4T1 and Caco-2 cells, Healthy rats	- ↑ 10.70-fold in plasma AUC compared to non-targeted DTX-lip - GSH responsive release at tumor	[127]
	Targeting ASBT in the distal ileum	DSLN-CSG	Docetaxel	Class 2	Lymph fistula rat model, Healthy rats and tumor bearing mice	- ↑ bioavailability by 5-fold compared to non-modified NPs - plasma DTX profile sustained up to 24 h - enhanced tumor growth inhibition and prevention	[128]
	Targeting VB_12_ mediated endocytosis	H/VC-LPN	Curcumin	CYP3A4 inhibitor	Caco-2/HT29-MTX co-culture, Healthy mice & rats	- mucus penetration - ↑ plasma AUC by 13.89 folds and C_max_ by 7.9 folds compared to curcumin suspension - multiple pathways absorption, including M cells and transcytosis across epithelium	[129]

^a^ Abbreviations: AM-SLNs: Asenapine maleate (AM) loaded solid lipid nanoparticles; AT-NLC: Atorvastatin (AT) nanostructured lipid carriers; CCN: Candesartan cilexetil (CC) loaded nanoemulsion; CLA-SLN: Clarithromycin (CLA) loaded solid lipid nanocarriers; Darunavir-SLN: Darunavir loaded solid lipid nanoparticles; DRD-SLN: Dronedarone hydrochloride (DRD) loaded solid lipid nanoparticles; DSLN-CSG: docetaxel (DTX)-loaded solid lipid nanoparticle (DSLN) coated with a glycocholic acid-chondroitin sulfate conjugate (CSG); DTX-ACSL-Lip: ACSL (Chitosan (CS) modified with acetic acid (A) and lipoic acid (L)) modified docetaxel (DTX) Liposomes; EFV-SLN: Efavirenz solid lipid nanoparticles; EuC-NLS: Eudragit-coated alendronate sodium (ALS) nanoliposomes; 5DN: 5-demethylnobiletin; GEN-loaded SLN: Genistein (GEN) loaded solid lipid nanoparticle; GSH: L-Glutathione; HACC-DTX-SLN: Hydroxypropyl trimethylammonium chloride chitosan (HACC) modified docetaxel-loaded solid lipid nanoparticles; H/VC-LPN: hydrophilic copolymer pHPMA associated with vitamin B_12_-grafted chitosan-modified lipid polymeric nanoparticles; IRSLNF3: Folic acid-grafted solid lipid nanoparticles (SLNs) bearing irinotecan hydrochloride trihydrate; LDL: Low-density lipoprotein; LPSN: WGA lectin conjugated paclitaxel (PTX) -loaded colloidal lipid nanostructures; NCC-SLN: N-carboxymethyl chitosan (NCC) coated curcumin-loaded solid lipid nanoparticles; pSLN: PEG2000-stearic acid modified solid lipid nanoparticle; SLN: Solid lipid nanoparticle; TC: Total cholesterol; TG: Triglyceride; VP16-NLCs: Etoposide (VP16) loaded nanostructured lipid carriers; ^b^ BDDCS class for each drug is obtained from Benet et al. [61].

## Supplementary Materials

The following are available online at https://www.mdpi.com/article/10.3390/pharmaceutics13081261/s1, Table S1: Source of the Log P value of various CYP3A4 interacting drugs.

## Abbreviations

API	Active pharmaceutical ingredient
AUC	Area under the plasma (blood) curves
BCS	Biopharmaceutics Classification System
BDDCS	Biopharmaceutics Drug Disposition Classification System
CAM	Complementary alternative medicine
CRASD	Controlled release amorphous solid dispersion systems
CYPs	Cytochrome P450
C_max_	Maximum (peak) drug concentration in the plasma, serum or whole blood
ER	Endoplasmic reticulum
F	the extent of drug bioavailability
GIT	Gastrointestinal tract
HMG-CoA	Hydroxyl-methyl-glutaryl coenzyme A
LNS	Lipid-based nanosystems
Microfold cells	M cells
NP(s)	Nanoparticle(s)
NLC	Nanostructured lipid carriers
NPs	nanoparticles
PK	pharmacokinetics
PLN	Polymer-lipid hybrid nanoparticles
PXR	Pregnane X receptor
SLN	Solid lipid nanoparticles
SJW	St. John wort
SNEDDS	Self-nanoemulsifying drug delivery systems
