# Supplementary material for: Nanoparticulate Drug Delivery Strategies to Address Intestinal Cytochrome P450 CYP3A4 Metabolism towards Personalized Medicine
Source: Pharmaceutics. 2021 Aug 16;13(8):1261. doi: 10.3390/pharmaceutics13081261 (PMC8399842; doi:10.3390/pharmaceutics13081261)
Supplement: Supplementary file 1 [file pharmaceutics-13-01261-s001.zip › pharmaceutics-1327296-supplementary.pdf]

pharmaceutics

Supplementary Materials: Nanoparticulate Drug Delivery Strategies to Address Intestinal Cytochrome P450 CYP3A4 Metabolism Towards Personalized Medicine

Ruixue Zhang [1], Ken Dong [2], Zhigao Wang [3], Ruimin Miao [1], Weijia Lu [1] and Xiaoyu Wu [2,*]

Table S1. Source of the Log P value of various CYP3A4 interacting drugs.

Drugs	Database [a]	Sources
Alprazolam	EPA DSSTox	https://comptox.epa.gov/dashboard/DTXSID4022577
Atorvastatin	HSDB	https://pubchem.ncbi.nlm.nih.gov/source/hsdb/7039
Carbamazepine	HSDB	https://pubchem.ncbi.nlm.nih.gov/source/hsdb/3019
Clarithromycin	HSDB	https://pubchem.ncbi.nlm.nih.gov/source/hsdb/8055
Cyclosporine	Drug Bank	https://www.drugbank.ca/drugs/DB00091
Dexamethasone	HSDB	https://pubchem.ncbi.nlm.nih.gov/source/hsdb/3053
Erythromycin	HSDB	https://pubchem.ncbi.nlm.nih.gov/source/hsdb/3074
Ethinyl estradiol	HSDB	https://pubchem.ncbi.nlm.nih.gov/source/hsdb/3587
Felodipine	DrugBank	https://www.drugbank.ca/drugs/DB01023
Fluconazole	EPA DSSTox	https://comptox.epa.gov/dashboard/DTXSID3020627
Indinavir	EPA DSSTox	https://comptox.epa.gov/dashboard/DTXSID4043802
Itraconazole	HSDB	https://pubchem.ncbi.nlm.nih.gov/source/hsdb/7839
Ketoconazole	HSDB	https://pubchem.ncbi.nlm.nih.gov/source/hsdb/7447
Lovastatin	HSDB	https://pubchem.ncbi.nlm.nih.gov/source/hsdb/6534

Midazolam	HSDB	https://pubchem.ncbi.nlm.nih.gov/source/hsdb/6751
Nicardipine	DrugBank	https://www.drugbank.ca/drugs/DB00622
Nifedipine	HSDB	https://pubchem.ncbi.nlm.nih.gov/source/hsdb/7775
Phenobarbital	HSDB	https://pubchem.ncbi.nlm.nih.gov/source/hsdb/3157
Phenytoin	HSDB	https://pubchem.ncbi.nlm.nih.gov/source/hsdb/3160
Ritonavir	HSDB	https://pubchem.ncbi.nlm.nih.gov/source/hsdb/7160
Saquinavir	HSDB	https://comptox.epa.gov/dashboard/DTXSID6044012
Sildenafil	HSDB	https://pubchem.ncbi.nlm.nih.gov/source/hsdb/7305
Simvastatin	HSDB	https://pubchem.ncbi.nlm.nih.gov/source/hsdb/7208
Tacrolimus	HSDB	https://pubchem.ncbi.nlm.nih.gov/source/hsdb/8195
Tadalafil	HSDB	https://pubchem.ncbi.nlm.nih.gov/source/hsdb/7303
Triazolam	HSDB	https://pubchem.ncbi.nlm.nih.gov/source/hsdb/6759
Vardenafil	HSDB	https://pubchem.ncbi.nlm.nih.gov/source/hsdb/7304
Verapamil	HSDB	https://pubchem.ncbi.nlm.nih.gov/source/hsdb/3928

a abbreviation: EPA DssTox: U.S. Environmental Protection Agency Distributed Structure-Searchable Toxicity; HSDB: Hazardous Substances Data Bank.
